# Insights into Infant Strongyloidiasis, Papua New Guinea

**DOI:** 10.3201/eid3109.241923

**Published:** 2025-09

**Authors:** Huan Zhao, Juciliane Haidamak, Eva Noskova, Vladislav Ilik, Barbora Pafčo, Rebecca Ford, Geraldine Masiria, Tobias Maure, Nichola Kotale, William Pomat, Catherine Gordon, Severine Navarro, Paul F. Horwood, Constantin Constantinoiu, Andrew R. Greenhill, Richard S. Bradbury

**Affiliations:** James Cook University, Townsville, Queensland, Australia (H. Zhao, J. Haidamak, P.F. Horwood, C. Constantinoiu, R.S. Bradbury); QIMR Berghofer Medical Research Institute, Brisbane, Queensland, Australia (J. Haidamak, C. Gordon, S. Navarro); Czech Academy of Sciences, Brno, Czech Republic (E. Noskova, V. Ilik, B. Pafčo); Masaryk University, Brno (E. Noskova, V. Ilik, B. Pafčo); Papua New Guinea Institute of Medical Research, Goroka, Papua New Guinea (R. Ford, G. Masiria, T. Maure, N. Kotale, W. Pomat); University of Queensland, Brisbane (C. Gordon); Federation University Australia, Gippsland, Victoria, Australia (A.R. Greenhill).

**Keywords:** Strongyloidiasis, *Strongyloides*, *Strongyloides fuelleborni*, parasites, infants, Papua New Guinea

## Abstract

The human-infecting parasite *Strongyloides fuelleborni* subspecies *kellyi* has been reported from the island of New Guinea. We analyzed fecal DNA extracts (n = 164) from 19 infants in Papua New Guinea by using *Strongyloides* real-time PCR and undertook metabarcoding of *cox1* and 18S rRNA hypervariable regions I and IV loci. Eight infants were infected with *Strongyloides* spp.; 7 were infected with *S. fuelleborni* subsp. *fuelleborni* and 1 with a *Strongyloides* sp. previously misattributed to *S. fuelleborni* subsp. *kellyi*. Phylogenetic and haplotyping analyses indicated *S. fuelleborni* in Papua New Guinea belongs to the Indochina subclade of *S. fuelleborni* subsp. *fuelleborni* and is not a unique subspecies. We report molecular evidence of *S. fuelleborni* subsp. *fuelleborni* infection in humans in the Pacific. Our findings also demonstrate the potential co-existence of an undescribed human-infecting *Strongyloides* sp. on the island of New Guinea, indicating a need for renewed clinical and epidemiologic investigations into infant strongyloidiasis.

New Guinea, consisting of Indonesian New Guinea in the west and Papua New Guinea in the east, is the world’s largest tropical island and a biodiversity hotspot. The island is home to the widespread and well-understood human pathogen *S. stercoralis* but also to a unique and enigmatic agent of human strongyloidiasis, the nematode *Strongyloides fuelleborni* subspecies *kellyi* (*1*–*3*). This *S. fuelleborni*–like intestinal nematode of humans was first reported in western Papua New Guinea in 1971 (*4*) and later found in Indonesian New Guinea (*5*). 

There was taxonomic confusion over the identity of the New Guinea *Strongyloides* and its relationship to *S. fuelleborni* from Africa. Unlike *S. stercoralis*, which is passed as rhabditiform larvae, *S. fuelleborni* subsp. *kellyi* is passed as eggs, resembling those of larvated hookworms, in feces (*1*,*6*). Viney et al. observed the adult nematodes were morphologically distinguishable by subtle differences in the peri-vulval cuticle of parasitic female specimens and the phasmidial pore position of free-living male specimens (*3*). A separate isoenzyme electrophoresis analysis revealed that the 2 worm isolates clustered closely, separate from other mammal-infecting *Strongyloides* spp. (*7*). On the basis of those findings, subspeciation of *S. fuelleborni* into 2 subspecies was proposed (*3*): *S. fuelleborni* subsp. *fuelleborni*, and *S. fuelleborni* subsp. *kellyi* for the New Guinea nematode. Of note, strains of *S. fuelleborni* from Asia were not included in that analyses (*3*,*7*).

The epidemiology of *S. fuelleborni* subsp. *kellyi* nematodes is similarly enigmatic. Infection in children has been observed within 3 weeks after birth (*6*). One study found the incidence of infection rose rapidly in the first 12 months of life and then stabilized until 5 years of age, at which age-related incidence began to drop (*8*). How infection occurs in children so young is unclear. Transmammary transmission has been suggested (*6*), although a screening of breastmilk from 40 mothers in an endemic village during the 1970s revealed no larvae (*9*). Nonhuman primates, the animal reservoir of *S. fuelleborni* subsp. *fuelleborni*, are not native to New Guinea, and attempts to find an animal reservoir for *S. fuelleborni* subsp. *kellyi* by screening domestic animals, including pigs, chickens, and dogs from villages where infections occurred at high prevalence in humans, were unsuccessful (*4*,*7*).

Heavy *S. fuelleborni* subsp. *kellyi* infection has been implicated as the cause of swollen belly syndrome (SBS), a rapidly fatal disease in infants around 2 months of age (*6*). SBS is a protein-losing enteropathy characterized by eosinophilia, distended abdomen, diarrhea, and respiratory distress (*10*). The etiology of SBS remains poorly understood. SBS was observed during 1974–1983, predominantly in 2 remote villages in mountainous regions of Papua New Guinea, Kanabea (Gulf Province, ≈1270 m above sea level) and Wanuma (Madang Province, ≈750 m above sea level). Only occasional, sporadic cases were reported elsewhere (*6*). In Kanabea, infection with an egg-producing *Strongyloides* sp. was found in 100% of infants 3–8 weeks after birth, with a mean average egg burden of 94,000 eggs/mL of feces (*9*). SBS caused 8% of infant deaths in this village until specific anthelmintic therapy was introduced (*6*). This possible correlation of *Strongyloides* sp. infection to SBS is despite reports of high burdens and prevalence of *Strongyloides* eggs, presumed to be *S. fuelleborni* subsp. *kellyi*, in infants across several other regions of Papua New Guinea, where infections were associated with nutritional deficiencies but not SBS (*11*,*12*). An unknown factor was proposed to be involved in the development of SBS (*10*).

We found only 1 *S. fuelleborni* subsp. *kellyi* DNA sequence recorded to date (*13*), a 330-bp segment of 18S (small subunit) rRNA that covers the hypervariable region (HVR) I (*14*). Phylogenetic analysis on the basis of this region placed *S. fuelleborni* subsp. *kellyi* in a clade with *S. cebus*, *S. papillosus*, and *S. venezuelensis* but distant from *S. fuelleborni* subsp. *fuelleborni*. That work prompted calls to elevate *S. fuelleborni* subsp. *kellyi* to the species rank (*2*). However, a criticism of that finding is that 18S rRNA HVR-I is a poor marker for inferring taxonomic positions of *Strongyloides* spp (*15*,*16*). That gene region did not enable the separation of *Strongyloides* spp. with spiraled and straight ovary morphotypes, whereas nearly full-length 18S rRNA and 28S rRNA sequences did (*13*,*17*). Furthermore, 1 study (*13*) found *S. fuelleborni* subsp. *fuelleborni* to be phylogenetically closer to *S. stercoralis* than to *S. papillosus*, contradicting evidence suggested by mitochondrial genome data (*18*). Molecular taxonomy of *Strongyloides* spp. determined by using markers such as mitochondrial cytochrome oxidase subunit 1 (*cox*1) and 18S rRNA HVR-IV showed considerable consistency with whole-genome and mitochondrial genome analyses (*18*,*19*). The true phylogeny and taxonomic identity of *S. fuelleborni* subsp. *kellyi* could be elucidated by using those informative markers.

Despite its public health significance, *S. fuelleborni* subsp. *kellyi* remains a neglected and underexplored human helminth. This enigmatic causative agent of strongyloidiasis in New Guinea warrants focused research to clarify its identity and epidemiology. We analyzed fecal DNA extracts from 19 infants in Papua New Guinea to explore the identity of *S. fuelleborni* subsp. *kellyi* infection.

## Methods

### Sampling

**Figure 1:**
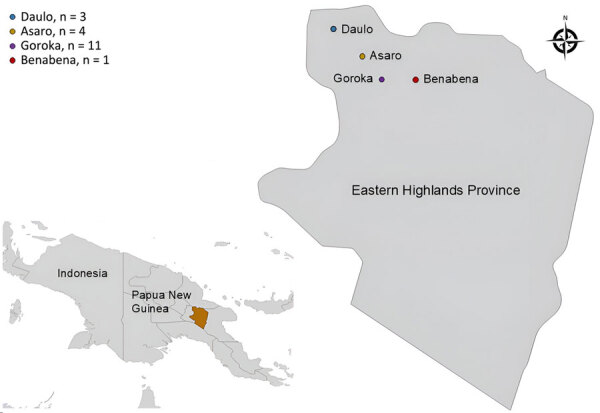
Sampling locations from study of infant strongyloidiasis, Eastern Highlands Province, Papua New Guinea. Participant numbers are shown for each location. Inset shows location of study area on the island of New Guinea.

We obtained samples from a 1-year longitudinal study investigating gut health in infants in Eastern Highlands Province, Papua New Guinea, during 2018–2019 (Figure 1). Ethics approval was obtained from the Papua New Guinea Institute of Medical Research Institutional Review Board (approval no. 1614) and the Papua New Guinea Medical Research Advisory Committee (approval no. 17.17). Infants were enrolled into the study within the first 5 weeks after birth. Initially, parents gave consent for participation in the study during antenatal clinic visits; after birth, informed consent was obtained from >1 parent or legal guardian. Fecal samples were collected from participants monthly for 12 months. 

For our study, we investigated the presence of *Strongyloides* spp. in fecal samples from 19 total infants, 12 boys and 7 girls. For samples used in our study, infants were 0–101 days (average 30 days) of age when their first fecal sample was collected. Clinical data on the participants was limited, but no marked gastrointestinal pathology was noted during the study.

Fresh fecal samples were transported to the Papua New Guinea Institute of Medical Research (Goroka, Papua New Guinea), where they were stored at −70°C. Samples were subsequently sent to Queensland Institute of Medical Research-Berghofer Medical Research Institute (Herston, Queensland, Australia) and James Cook University (Townsville, Queensland, Australia) for laboratory analysis. We extracted DNA from each sample by using a QIAamp DNA mini kit (QIAGEN, https://www.qiagen.com). Before extraction, we mixed fecal samples (≈200 mg) with 500 µL of rapid 1-step extraction buffer, 15 µL of proteinase K, and 500 µL of 0.5-mm silica/zirconia beads (Daintree Scientific, http://www.daintreescientific.com.au) in 2-mL screw-cap tubes (Starstedt, https://www.sarstedt.com). We homogenized the tubes at 6,500 rpm for 40 seconds by using a Precellys homogenizer (Bertin Technologies, https://www.bertin-technologies.com). After homogenization, we incubated the samples at 95°C for 5 minutes and then centrifuged at 4,000 relative centrifugal force for 3 minutes. We transferred the supernatant to a new 1.5-mL tube and then followed the QIAGEN protocol for DNA extraction.

We assessed the extracted fecal DNA for quality and quantity by using a NanoDrop 2000 (Thermo Fisher Scientific, https://www.thermofisher.com). We used DNA samples with a concentration >10 ng/µL, and a 260/280 ratio between 1.6 and 2.10 as quantitative PCR templates. We reextracted 5 samples that did not meet those requirements; all subsequently reached the required DNA concentration. We then diluted the DNA 1:5 with Milli Q water (Thermo Fisher Scientific) and subjected it to 2 *Strongyloides* qPCRs (*20*,*21*). We confirmed positive qPCR results by triplicate testing and samples were considered positive when the cycle threshold (Ct) was <35. To tentatively infer the identity of the qPCR product, we conducted Sanger sequencing of positive amplicons from the 2 qPCRs, targeting a 471-bp region of 18S rRNA (*20*) and a 138-bp repeat sequence (*21*) by using the BigDye Direct Cycle Sequencing kit (Thermo Fisher Scientific).

We also performed a metabarcoding assay targeting 18S rRNA HVR-IV (≈252-bp) by using the Illumina MiSeq Reagent Nano Kit v2 (Illumina, https://www.illumina.com) (*16*). We then subjected positive samples to additional metabarcoding of the 18S rRNA HVR-I (≈432-bp) and partial *cox*1 (217-bp) genotyping targets (*16*) by using the same Illumina MiSeq platform. We conducted sequencing with 500 cycles (paired-end, 250-bp) for 18S rRNA products and 300 cycles (paired-end, 150-bp) for the *cox*1 products to ensure adequate sequencing coverage.

### Statistical and Bioinformatic Analysis

We imported demographic and qPCR data into Excel (Microsoft, https://www.microsoft.com) for statistical analysis. We had the raw Illumina sequencing data analyzed by 3 blinded researchers at James Cook University and the Institute of Vertebrate Biology (IVB) through 2 different computational pipelines: a custom Geneious Prime (https://www.geneious.com) workflow as previously published (*16*) at both James Cook University and IVB, and a combination of Skewer (*22*) and DADA2 (*23*) packages implemented in R software version 4.2.2 (The R Project for Statistical Computing, https://www.r-project.org) at IVB. Both pipelines incorporated read quality control, contig assembly, and haplotype assignment. We conducted maximum-likelihood phylogenetic analysis in MEGA 11 (https://www.megasoftware.net) and Bayesian inference phylogenetic analysis in MrBayes (https://nbisweden.github.io/MrBayes) on MUSCLE-aligned (https://github.com/rcedgar/muscle) *cox1* sequences by using the general time-reversible model for nucleotide substitutions. We then used Cohen’s κ to assess the diagnostic agreement between different molecular methods for the detection of *Strongyloides* spp.

## Results

**Figure 2:**
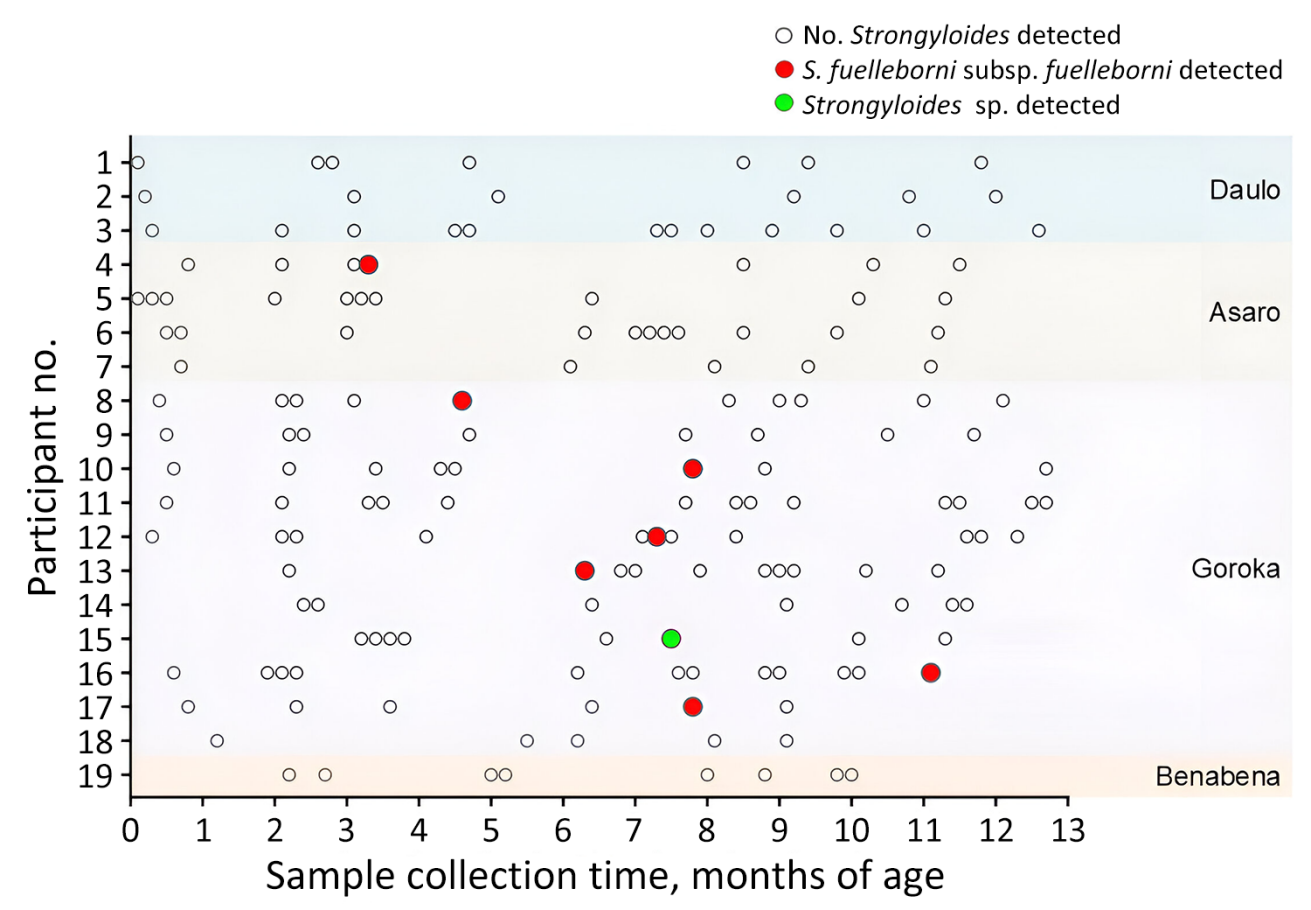
Sampling frequency and *Strongyloides* infection in infants from Eastern Highlands Province, Papua New Guinea. At least 5 samples were collected from each infant enrolled during the first 13 months of life. Samples that demonstrated molecular evidence of *Strongyloides* spp. infection are shown. Sample locations are indicated at right.

A total of 164 fecal samples were collected from 19 infants during 5–13 occasions over the study period (Figure 1, 2). Eight (42%) of the 19 infants, comprising 6 boys and 2 girls, were found to be infected with a *Strongyloides* spp. Infections were detected at an average age of 212 days (range 94–334 days) (Figure 2). Of the 8 positive cases, fecal metabarcoding at *cox1* and 18S rRNA HVR-I and HVR-IV loci identified 6 infections as *S. fuelleborni* subsp. *fuelleborni* and 1 as a *Strongyloides* sp. previously attributed to *S. fuelleborni* subsp. *kellyi* (*13*) (Table). The remaining positive sample could only be amplified at the qPCR 18S rRNA target. That 471-bp sequence was 99.2% identical to a published *S. fuelleborni* subsp. *fuelleborni* sequence (GenBank accession no. AB272235) by BLAST analysis (https://blast.ncbi.nlm.nih.gov/).

**Table:** *Strongyloides* samples analyzed and their genotypes in study of infant strongyloidiasis, Papua New Guinea*

Sample no.	Patient age, d/sex	Species detected	18S rRNA HVR-IV haplotypes	18S rRNA HVR-I haplotypes	*cox*1 haplotypes
1	205/M	*S. fuelleborni* subsp. *fuelleborni*	S (PQ774622)	XIV (PV489783)	3 OTUs (PQ774615–7)
2	94/M	*S. fuelleborni* subsp. *fuelleborni*	S (PQ774622)	XIV (PV489783)	3 OTUs (PQ774615–7)
3	140/F	*S. fuelleborni* subsp. *fuelleborni*	S (PQ774622)	XIV (PV489783)	2 OTUs (PQ774615, PQ774616)
4	234/M	*S. fuelleborni* subsp. *fuelleborni*	S (PQ774622)	XIV (PV489783)	2 OTUs (PQ774615, PQ774616)
5	227/F	*Strongyloides* spp.	1 OTU (PQ774624)	3 OTUs (PV489780–2)	NA†
6	225/M	*S. fuelleborni* subsp. *fuelleborni*	S (PQ774622)	XIV (PV489783)	2 OTUs (PQ774616, PQ774617)
7	236/M	*S. fuelleborni* subsp. *fuelleborni*	S (PQ774622)	XIV (PV489783)	2 OTUs (PQ774615, PQ774616)
8	334/M	*S. fuelleborni* subsp. *fuelleborni*	NA‡	NA‡	NA‡

*Values in parentheses are GenBank accession numbers. HVR, hypervariable region; NA, not available; OTU, operational taxonomic unit. †*cox1* region cPCR did not amplify. ‡Metabarcoding on the 18S rRNA HVR-IV locus yielded no detectable product on agar electrophoresis.

**Figure 3:**
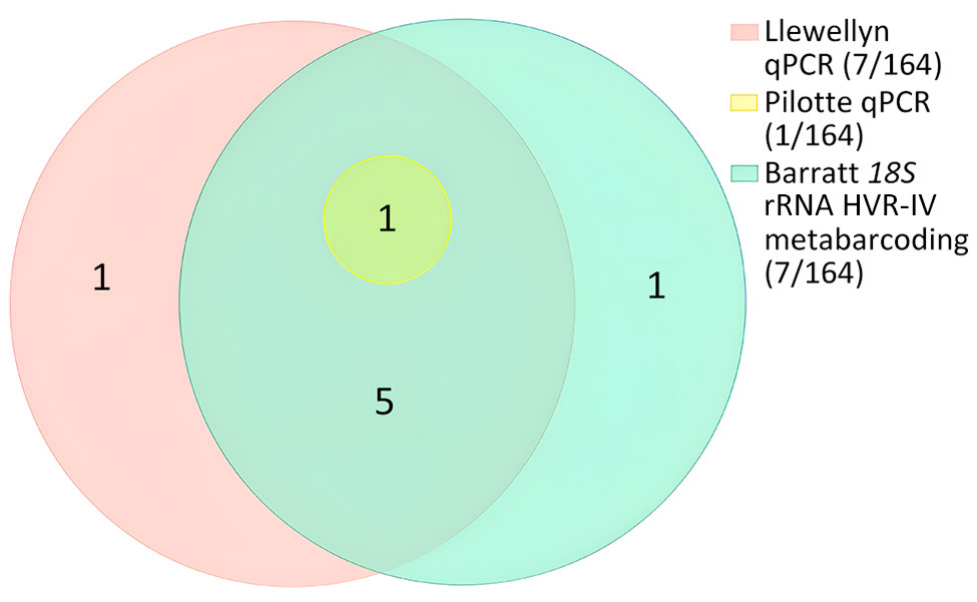
Euler diagram showing the performance of 2 qPCRs (*20*,*21*) and 18S rRNA HVR-IV metabarcoding (*16*) for the detection of *Strongyloides* spp. in 164 infant fecal samples from Papua New Guinea. Values in parentheses are no. positive samples/total no. tested. HVR, hypervariable region; qPCR, quantitative PCR.

The previously published genus-specific qPCR method (*20*) performed comparably with a previously published 18S rRNA HVR-IV metabarcoding method (*16*) in detecting *Strongyloides* infections, yielding a Cohen’s κ coefficient of 0.85. The second qPCR method (*21*) did not detect *S. fuelleborni* subsp. *fuelleborni* but did detect the *Strongyloides* spp. previously considered *S. fuelleborni* subsp. *kellyi* (Figure 3).

**Figure 4:**
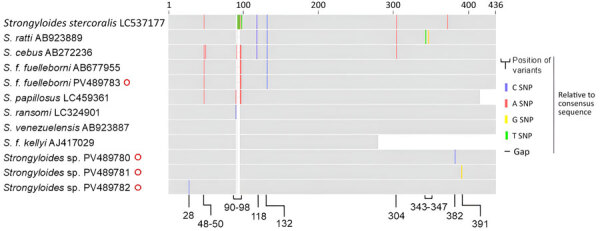
Schematic of MUSCLE-aligned (https://github.com/rcedgar/muscle) *Strongyloides* sp. *18S rRNA* HVR-I sequences from study on infant strongyloidiasis, Papua New Guinea. Sequences obtained are marked with red circles. Previously published sequences are annotated with their GenBank accession numbers. *S. f.* *fuelleborni*, *S.* *fuelleborni* subsp. *fuelleborni*; *S. f.* *kellyi*, *S.* *fuelleborni* subsp. *kellyi*.

We obtained sequences of 18S rRNA HVR-I (432-bp) and HVR-IV (≈252-bp) from 7 samples (Table). Six positive samples harbored HVR-IV haplotype S and HVR-I haplotype XIV, both genotypes previously identified in all *S. fuelleborni* subsp. *fuelleborni* isolates from Asia. The remaining positive sample was infected with 3 HVR-I haplotypes (deposited into GenBank under accession nos. PV489780–2): two with 275-bp sequences identical to the only published sequence of *S. fuelleborni* subsp. *kellyi* (GenBank accession no. AJ417029) (*13*) and the third differing by 1 single-nucleotide polymorphism (SNP) (T-C at position 28). HVR-I sequences of that *Strongyloides* sp. (432-bp) differed from those of *S. ransomi* (GenBank accession nos. LC324901, OP288111) by 2 SNPs and from *S. venezuelensis* (GenBank accession no. AB923887) by 1 SNP (Figure 4). At the HVR-IV locus, the 248-bp sequence was 100% identical to sequences of *S. ransomi* (GenBank accession nos. OP288111, KU724127) and *S. venezuelensis* (GenBank accession no. AB923887).

**Figure 5:**
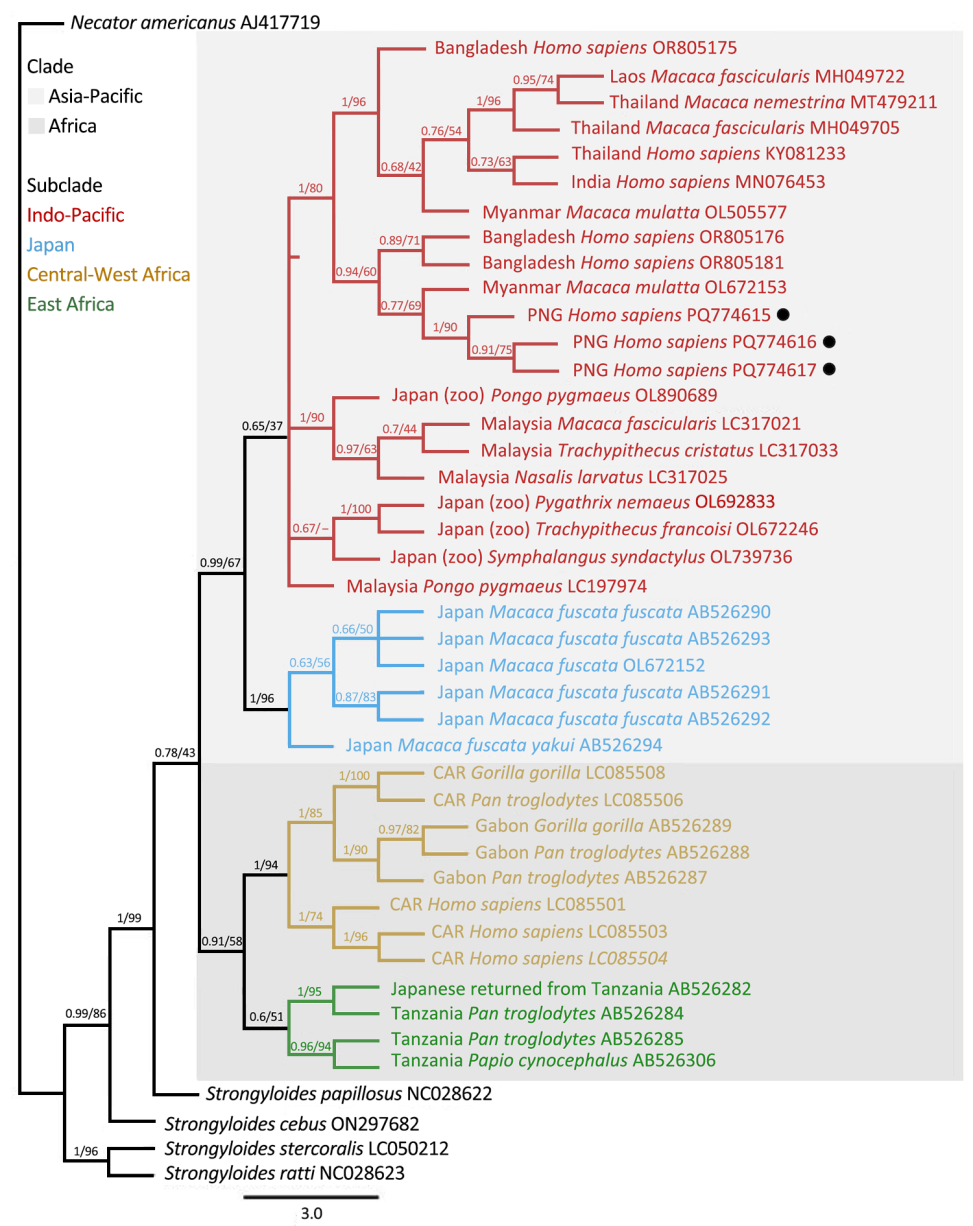
Phylogenetic tree of *Strongyloides fuelleborni* subsp. *fuelleborni* on the basis of *cox*1 sequences from study on infant strongyloidiasis in Papua New Guinea. Bayesian posterior probability and maximum-likelihood bootstrap support percentages (1,000 replicates) are indicated at the nodes. Black dots indicate sequences obtained in this study. Previously published sequences are annotated with their country of origin, host species, and GenBank accession numbers. Clades and subclades of *S. fuelleborni* subsp. *fuelleborni* are color-coded on the basis of geographic regions. Scale bar indicates number of substitutions per site. –, no statistics are available; CAR, Central African Republic; PNG, Papua New Guinea.

*cox1* sequences were available for 6 samples, all assigned to *S. fuelleborni* subsp. *fuelleborni* (Table). The seventh sample, containing the genetically distinct *Strongyloides* sp., did not amplify. We identified 3 separate *S. fuelleborni* subsp. *fuelleborni* haplotypes (deposited into GenBank accession nos. PQ774615–7). Maximum-likelihood and Bayesian inference phylogenetic analyses on the *cox1* locus placed *S. fuelleborni* subsp. *fuelleborni* from Papua New Guinea in a clade with *S. fuelleborni* subsp. *fuelleborni* from Myanmar *rhesus macaques* (GenBank accession no. OL672153). Those sequences also clustered closely with *S. fuelleborni* subsp. *fuelleborni* from Bangladesh (GenBank accession nos. OR805176 and OR805181) (Figure 5).

## Discussion

We provide molecular evidence of human infections with *S. fuelleborni* subsp. *fuelleborni* outside of Asia and Africa. The potential existence of an undescribed human-infecting *Strongyloides* sp., previously misattributed to *S. fuelleborni* subsp. *kellyi* (*13*), is also suggested. On the basis of those findings, we hypothesize that >2 genetically distinct, non–*S. stercoralis Strongyloides* nematodes infect humans in Papua New Guinea and that *S. fuelleborni* subsp. *kellyi*, as previously described (*3*), is not a unique subspecies but is likely synonymous with the Asia-Pacific clade of *S. fuelleborni* subsp. *fuelleborni*.

Our phylogenetic findings corroborate a previously published study (*3*) that described very subtle morphologic distinctions between *S. fuelleborni* adult isolates from Africa and Papua New Guinea. Both strains differed markedly in morphology from *S. ransomi* in Papua New Guinea pigs. Because those analyses did not include isolates of *S. fuelleborni* subsp. *fuelleborni* from Asia, and no morphologic studies of this nematode from Asia exist, it is possible that study described the first representatives of what is now recognized as the clade of *S. fuelleborni* subsp. *fuelleborni* from Asia (*3*). Further comparative morphologic and genomic analyses of adult isolates from Africa, Asia, and the Pacific are required to test that hypothesis. If confirmed, the finding would warrant a revision of current subspecies epithets to reflect the divergence between Africa and Asia-Pacific *S. fuelleborni*.

In Africa and Asia, *S. fuelleborni* subsp. *fuelleborni* is a common infection of nonhuman primates and is considered a zoonosis originating from those animals (*24*,*25*). Multiple studies suggest human-to-human transmission of *S. fuelleborni* subsp. *fuelleborni* occurs in some regions of Africa (*25*,*26*). This suggestion is supported by genetic analysis (*27*) identifying a human-specific subpopulation among *S. fuelleborni* subsp. *fuelleborni* isolates from Africa. Because of the absence of a nonhuman primate reservoir on the island of New Guinea, it is likely that *S. fuelleborni* subsp. *fuelleborni* has adapted to exclusive human-to-human transmission after being introduced to Papua New Guinea through human migration (*28*).

The distinct *Strongyloides* genospecies identified in 1 infant from this study corresponds to the genospecies previously misattributed to *S. fuelleborni subsp. kellyi* (*13*). This species was indistinguishable from *S. ransomi* and *S. venezuelensis* at the 18S rRNA HVR-IV locus and exhibited only 1–2 SNPs at the HVR-I locus, suggesting a recent common ancestry among them. An earlier isoenzyme electrophoretic analysis (*7*) found that 4 of 26 *Strongyloides* isolates recovered from persons in Papua New Guinea clustered closely with *S. ransomi* from local pigs. Although there was speculation those samples might have originated from pigs, it is plausible they represented the same distinct species identified in this and a previous study (*13*). However, caution is warranted when interpreting taxonomic placements based solely on single-locus 18S rRNA data from fecal DNA, without accompanying morphologic data, because those inferences might be artifactual. Detailed morphologic analysis of adult isolates, combined with whole-genome or mitochondrial genome sequencing, is needed to resolve the taxonomic status of this nematode.

Historical reports of *S. fuelleborni* subsp. *kellyi* predated the molecular era and relied solely on microscopic identification of *Strongyloides* eggs (*4*,*6*,*8*,*9*,*12*,*28*). Those data require reassessment because they might conflate infections with 2 co-endemic human-infecting *Strongyloides* spp. nematodes. Surveys conducted during 1976–1997 in Papua New Guinea reported *S. fuelleborni* subsp. *kellyi* prevalence of 20%–93% in children, with rates reaching 60% within the first year of life (*6*,*8*,*9*,*12*,*28*). We similarly found a high incidence of *S. fuelleborni* infection (7/19), but also 1 infection (1/19) with an undescribed *Strongyloides* sp. in Papua New Guinea infants. Future surveillance for strongyloidiasis in New Guinea should use species-specific molecular tools to differentiate those 2 agents.

Our findings provide explanation for much of the unknown epidemiology of infant strongyloidiasis in Papua New Guinea. Patent *S. fuelleborni* infections in infants as young as 18 days of age have been found in Papua New Guinea (*6*). Sampling of breastmilk from mothers in Papua New Guinea (*29*) did not identify any *Strongyloides* larvae; however, difficulties in obtaining fecal samples from those mothers left their infection status uncertain. In the same village, the prevalence of *Strongyloides* spp. in adult feces was only 14% (*12*). In a survey of 25 lactating mothers of infants with confirmed *S. fuelleborni* subsp. *fuelleborni* infection in the Democratic Republic of the Congo, *Strongyloides* spp. filariform larvae were found in the breast milk of 1 mother (*30*). That finding suggests transmammary transmission could be responsible for the high rate of infection in infants as young as 50–74 days of age in that region (*30*). Because our genetic analysis indicates the worms we identified belong to clades of the same species, it is plausible that transmammary transmission to Papua New Guinea infants might occur. That speculation warrants further investigation, and the use of molecular genotyping tools might be necessary to track transmission patterns.

The attribution of *S. fuelleborni* subsp. *kellyi* as the causative agent of infantile SBS requires further validation. Despite *S. fuelleborni* infection being widespread in children in some parts of Africa, SBS has not been reported from that continent (*25*). Neither has SBS associated with *S. fuelleborni* infection been documented in Asia. The detection of a *Strongyloides* genospecies closely related to *S. ransomi* of pigs raises an alternative hypothesis regarding the etiology of SBS in Papua New Guinea. In newborn suckling piglets, *S. ransomi* infection causes a protein-losing enteropathy characterized by villus atrophy, malabsorption, diarrhea, progressive dehydration, hypoproteinaemia, anemia, anorexia, emaciation, sudden death (*31*,*32*), and reduced hepatic protein synthesis (*33*), a clinical picture strikingly similar to SBS in infants from Papua New Guinea (*1*,*6*). Because of the substantial genetic similarity observed between *S. ransomi* and this genospecies, a shared pathogenic mechanism is plausible. *S. ransomi* detection in only 1 of 19 infants also mirrors the rare and sporadic occurrence of SBS previously reported in this population (*6*,*9*,*10*). Further research is needed to explore this hypothesis and clarify the etiology and epidemiology of SBS.

In our study, not all diagnostic *Strongyloides* spp. qPCRs detected *S. fuelleborni* infection. The Llewelyn modification (*20*) of Verweij (*34*) qPCR appears generic and detected all except 1 *S. fuelleborni* infection. In contrast, the Pilotte *S. stercoralis* qPCR (*21*) only amplified DNA of the undescribed *Strongyloides* sp. and did not detect *S. fuelleborni* infections. That finding demonstrates that the specificity of PCR diagnostics used must be considered in future surveillance of *Strongyloides* spp. infections in humans and animals, because the choice of qPCR might markedly affect the findings of any survey.

In summary, we present molecular evidence of human infections with *S. fuelleborni* subsp. *fuelleborni* nematodes in Papua New Guinea. On the basis of this evidence and existing morphologic data on *S. fuelleborni* subsp. *kellyi*, we hypothesize that *S. fuelleborni* subsp. *kellyi* is not a unique subspecies but rather represents *S. fuelleborni* subsp. *fuelleborni* Asia-Pacific clade infections occurring in humans in New Guinea. We further molecularly identified an undescribed *Strongyloides* species in 1 infant from Papua New Guinea and raise questions about the possible role of this undescribed species in the etiology of infantile SBS. Renewed clinical, epidemiologic and taxonomic investigations into infant strongyloidiasis in this region are needed to increase clinician awareness of such infections and guide prevention and treatment efforts.
